# Differences in DNA damage repair gene mutations between left‐ and right‐sided colorectal cancer

**DOI:** 10.1002/cam4.5716

**Published:** 2023-04-25

**Authors:** Wei Huang, Wenliang Li, Ning Xu, Hui Li, Zihan Zhang, Xiaolong Zhang, Tingting He, Jicheng Yao, Mian Xu, Qingqing He, Lijie Guo, Sen Zhang

**Affiliations:** ^1^ Department of Colorectal & Anal Surgery The First Affiliated Hospital of Guangxi Medical University Nanning China; ^2^ Department of Colorectal Surgery Yunnan Cancer Hospital Yunnan China; ^3^ Department of Oncology The First Affiliated Hospital of Kunming Medical University Yunnan China; ^4^ Shanghai OrigiMed Co., Ltd Shanghai China

**Keywords:** DDR mutations, genomic landscape, MSS CRC, prognosis

## Abstract

**Background:**

Colorectal cancer (CRC) is the third leading cause of cancer‐related deaths worldwide. Studies have shown that the DNA damage response (DDR) mutation is strongly associated with microsatellite instability (MSI) status and is an indication for patients with CRCs receiving immune checkpoint inhibitor (ICI) treatment. However, DDR mutation in microsatellite stable (MSS) CRC remains unclear.

**Methods:**

In this study, Fisher's exact test, Student'st‐test, Wilcoxon rank‐sum test and Cox proportional hazards regression model were performed, and a *p* value of < 0.05 was considered statistically significant.

**Results:**

The most common gene alterations were *APC* (77%), *TP53* (73%), *KRAS* (48%), and *PIK3CA* (25%). The mutationfrequency of *APC* and *TP53* in left‐sided CRC was significantly higher than that for right‐sided CRC, while the mutation frequency of *PIK3CA, ACVR2A, FAT4,* and *RNF43* in right‐sided CRC was significantly higher than that for left‐sided CRC. DDR mutations occurred in100% of MSI CRCs and in 83.77% of MSS CRCs, with the most frequently mutated DDR genes being *ARID1A* (7.5%), *ATM* (5.7%,) and *BRCA2* (2.6%). When right‐ and left‐sided CRCs were compared, no significant difference was observed for DDR genes and pathways. A survival analysis indicated that the DDR mutation was not associated with overall survival (OS) in MSS CRCs, while left‐sided patients with homologous recombination repair (HRR) pathway mutations had a significantly prolonged OS compared with right‐sided CRCs.

**Conclusions:**

Here, we found that stage and grade were statistically significant independent prognostic factors in the left‐sided CRC and the right‐sided CRC, recommending treatment for these patients stratified by stage. For the future, utilizing DDR gene defects for expanding treatment options and improving prognosis is an issue worth exploring.

## Graphical abstract

In this study, we utilized next generation sequencing (NGS) to investigate genomic alterations in colorectal cancer (CRC), with a particular emphasis on comparing DNA damage response (DDR) mutations in left‐ and right‐sided CRC. Furthermore, we assessed the correlation between DDR mutations and prognosis for microsatellite stable (MSS) CRC.

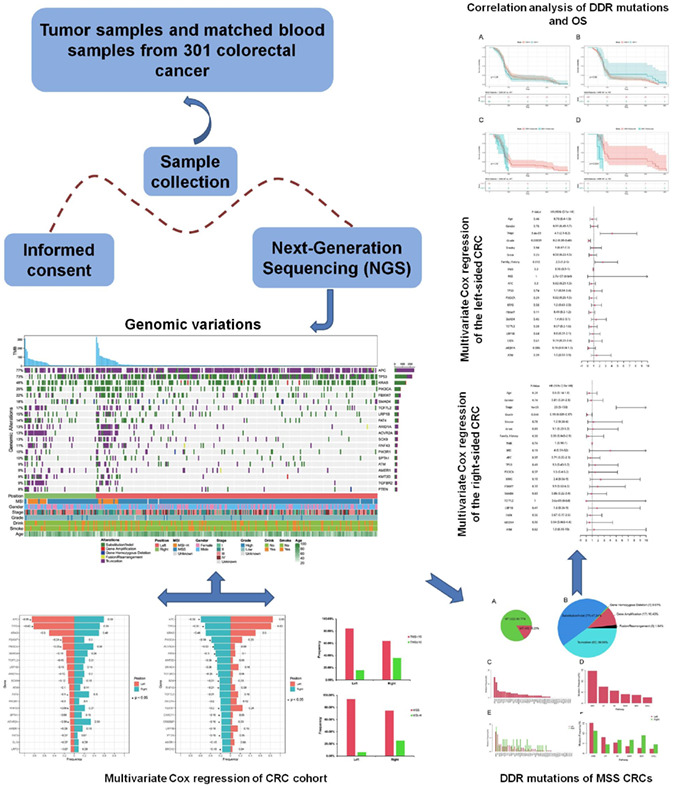

## 1 INTRODUCTION

Colorectal cancer (CRC) is a malignancy characterized by the abnormal growth of large intestine tissue.^1^ CRC is one of the most common cancers, with an incidence rate that ranks third in the world, and is more prevalent in men than in women.^2^ Despite effective cancer screening technology and modern medicine, the incidence and mortality of CRC have both increased in China.^3^ Therefore, identifying novel diagnostic and prognostic biomarkers and exploring potentially relevant targets for the treatment of CRC are important goals.

Due to the need for further research, studies are currently being conducted on genomic alterations in the DNA damage response (DDR) pathway. In this context, DDR genes mutations are emerging as novel targets for cancer therapy. The DDR pathway's function is to accurately correct and repair DNA damage in a timely manner in order to preserve cell genome integrity, so as to inhibit cell aging, apoptosis, and carcinogenesis, and to ensure normal life activities.^4^, ^5^ Based on DNA lesions, DDR comprises eight pathways: mismatch repair (MMR), base excision repair (BER), nucleotide excision repair (NER), homologous recombination repair (HRR), nonhomologous end‐joining (NHEJ), checkpoint factors (CPF), Fanconi anemia (FA), and translesion DNA synthesis (TLS).^6^ DDR deficiencies in many cancers offer new opportunities for targeted, precision therapy. Poly (ADP‐ribose) polymerase‐inhibitors (PARPi) are currently applied for the treatment of HRR (BRCA1/2, BRD4, PTEN, or other HRR related genes) defective cancers such as ovarian cancer,^7^ pancreatic cancer,^8^ and prostate cancer.^9^ Additionally, once a failure to maintain genomic integrity and stability is established, DDR alterations may induce a hyper‐mutated phenotype with a higher tumor mutation burden (TMB) or a microsatellite instability‐high (MSI‐H) status, established as a predictive biomarker for clinical benefit from immune checkpoint inhibitor (ICI) treatment.^10^, ^11^ For instance, Wang et al.^12^ revealed that mutations within the DDR pathways of HRR‐MMR or HRR‐BER were associated with increased TMB, neoantigen load, and increased levels of immune gene expression signatures and served as potential predictors of superior survival outcomes in response to immune checkpoint blockades.^12^

In CRC, the role of DDR alterations is still widely unknown and data regarding their clinical impacts are scarce. In recent years, a subset of studies has revealed germline and/or DDR defects in CRC, with a prevalence between 13.8% and 36%.^13^, ^14^, ^15^ Regardless of MSI status, the median (mTMB) of CRC with DDR alterations was found to be higher, as well as the positive rate of PD‐L1.^15^ Additionally, DDR mutations have been correlated with improved overall survival (OS) in CRCs treated with ICIs.^13^ A recent study indicated that DDR‐related *ATM* or *BRCA2* somatic mutations are promising biomarkers for assessing the response of stage III CRC patients to oxaliplatin‐based chemotherapy.^16^ However, at present, there is a lack of studies that systematically compare DDR mutations between left‐ and right‐sided CRC, and little is known about the prognostic impact of DDR mutations in microsatellite stable (MSS) CRC patients.

Therefore, the present study systematically compared DDR mutations between left‐ and right‐sided CRC and investigated the correlation between DDR mutations and prognosis for MSS CRC.

## 2 MATERIALS AND METHODS

### 2.1 Patients and tumor samples

Tumor samples from 301 CRC patients were collected at The First Affiliated Hospital of Guangxi Medical University and The First Affiliated Hospital of Kunming Medical University Hospital from 2014 to 2019. Pathological sections were cut from formalin‐fixed paraffin‐embedded (FFPE) tumor blocks for subsequent use. To confirm that samples contained the highest tumor cell purity (>50%), prior to sequencing, FFPE tumor samples were evaluated by pathologists. All patients signed written informed consent for the collection and use of tumor samples. This study was approved by the Ethics Committee of The First Affiliated Hospital of Guangxi Medical University.

### 2.2 Identification of genomic alterations and tumor mutational burden (TMB)

Formalin‐fixed, paraffin‐embedded (FFPE) tumor tissues and matched blood samples were obtained from the First Affiliated Hospital of Guangxi Medical University. At least 50 ng of cancer tissue DNA was extracted from the 40 mm FFPE and from blood samples using a DNA Extraction Kit (QIAamp DNA FFPE Tissue Kit, Qiagen) for subsequent targeted NGS‐based genomic testing (OrigiMed). Genomic mutations were detected using the NGS‐based YuanSu™ (OrigiMed) gene panel, which covers all coding exons for 450 cancer‐related genes frequently altered in solid tumors (including the 45 DDR genes). Genes were captured and sequenced, with a mean depth of 800× and with a minimum depth of coverage of ≥200×, using an Illumina NextSeq 500 (Illumina) by following the steps described in Frampton et al.^17^ The quality scores of ≥40 were used for this study. Mutational variant allele frequency (VAF) was defined as the number of variant reads divided by the number of total reads and reported as a percentage. Mutations with VAF ≥1% were included for analysis.

Genomic alterations (GAs) were identified based on the described procedure of Cao et al.^18^ Single‐nucleotide variants (SNVs) were identified using MuTect (v1.7). Insertion–deletions (Indels) were identified using PINDEL (v0.2.5). The functional impact of GAs was annotated using SnpEff 3.0. Copy number variation (CNV) regions were identified with Control‐FREEC (v9.7), using the following parameters: window = 50,000 and step = 10,000. Gene fusions were detected using an in‐house developed pipeline. Gene rearrangements were assessed by employing the Integrative Genomics Viewer (IGV). TMB was measured by counting coding somatic mutations, including SNVs and Indels, per megabase of the sequence examined for each patient. Since cutoffs for categorizing the TMB status of CRC have not been defined, we used criteria established in a previous study for different tumor types.^19^ In this study, TMB‐L was defined as <10 mut(mutations)/Mb, and TMB‐H was defined as ≥10 mut/Mb of sequenced DNA.

### 2.3 Definition of DNA damage repair

To identify DDR inactivation mutation status, the DNA data of nonsynonymous copy number variants, single‐nucleotide variants, and multi‐nucleotide variants for 45 DDR genes (Table S1) were retrieved and combined. DDR pathway alternations were defined as any nonsynonymous somatic alteration (including missense, nonsense, insertion, deletion, and splice) in the protein‐coding region or the presence of homozygous deletions of at least one gene involved in the corresponding DDR pathways.

### 2.4 Statistical analyses

For statistical analyses, SPSS version 22.0 (SPSS Inc.) was applied. Fisher's exact test was used for the association analysis of categorical variables. Student's *t* test and Wilcoxon rank‐sum test were used for the association analysis of normally distributed data and nonnormally distributed data, respectively. A Kruskal–Wallis test was used for analyses of the association between multiple groups of nonparametric data. A Cox proportional hazards regression model was used for quantifying overall survival (OS). A *p* value of <0.05 was considered statistically significant.

## 3 RESULTS

### 3.1 Patient characteristics

For this study, a total of 301 CRC patients were recruited, of which 240 had a left‐sided CRC diagnosis and 61 had a right‐sided CRC diagnosis. One hundred and twenty‐one patients were younger than 55 years old, and 180 patients were older than 55. One hundred and twenty‐one (40.2%) of patients were females and 180 (59.8%) were males. Based on tumor stage, there were 33 (11.0%) patients at Stage I, 99 (33.0%) patients at Stage II, 120 (39.9%) patients at Stage III, and 47 (15.5%) patients at Stage IV. The tumor stage for two (0.6%) patients was unknown. The tumor for 280 (93.0%) patients was at low grade, 16 (5.3%) patients had high‐grade tumors, and tumor grade for the remaining 5 (1.7%) patients was unknown. Seventy‐one (23.6%) patients had a history of smoking, 64 (21.3%) had a history of alcohol consumption, and 59 (19.6%) had a family history. A follow‐up for the 301 patients indicated that 133 (41.2%) patients did not progress, 4 patients (1.3%) had a recurrence, 158 (52.5%) patients had metastasis, and 6 (2.0%) patients had no progression. At the last follow‐up, 188 (62.5%) patients survived, 51 (16.9%) died, and 62 (20.6%) patients had an unknown survival status. Sixty (20%) patients were defined as TMB‐H (TMB ≥10 mut/Mb), while 241 (80%) patients were defined as TMB‐L (TMB < 10 mut/Mb). The mTMB of right‐sided CRC was 7.7 muts/Mb, whereas the mTMB of left‐sided CRC was 5.4 muts/Mb. The frequency of TMB‐H in right‐sided CRC was higher than that in left‐sided CRC (36.1% vs. 15.8%, respectively, *p* < 0.001, Figure S1A). Additionally, 30 (10.2%) patients were defined as MSI‐H, 265 (88%) patients were defined as MSS, and 6 (2.0%) patients had an unknown MSI status. The frequency of MSI‐H in left‐sided CRC was 6.4%, while was 25.4% in right‐sided CRC (Figure S1B). The frequency of MSI‐H in right‐sided CRC was higher than that in left‐sided CRC (25.4% vs. 6.36%, respectively, *p* < 0.001). Detailed clinical characteristics for each patient are provided in Table 1.

**TABLE 1:** Clinical characteristics of the 301CRCs.

	Total	Left	Right	OR (95% CI)	*p* value
Age					
≤55	121	96	25	Reference	
>55	180	144	36	0.9601 (0.5229–1.783)	0.8849
Gender					
Female	121	91	30	Reference	
Male	180	149	31	0.6321 (0.3448–1.1587)	0.1432
Stage					
I	33	30	3	Reference	
II	99	77	22	2.8383 (0.7659–15.8919)	0.1253
III	120	93	27	2.8868 (0.7991–15.9107)	0.1351
IV	47	38	9	2.3448 (0.524–14.639)	0.3413
Unknown	2	2	NA		
Grade					
Low	280	224	56	Reference	
High	16	11	5	1.8139 (0.4744–5.9497)	0.3367
Unknown	5	5	NA		
Smoke					
No	230	182	48	Reference	
Yes	71	58	13	0.8503 (0.3942–1.7336)	0.7366
Drink					
No	237	187	50	Reference	
Yes	64	53	11	0.7768 (0.3403–1.6476)	0.5999
Family History					
No	233	194	39	Reference	
Yes	59	42	17	2.008 (0.9692–4.0531)	0.0422
Unknown	9	4	5		
Progress					
No	133	109	24	Reference	
Recurrent	4	2	2	4.472 (0.31–64.6123)	0.1629
Metastatic	158	124	34	1.2444 (0.6697–2.3401)	0.556
Unknown	6	5	1		
Survival					
Alive	188	147	41	Reference	
Dead	51	40	11	0.986 (0.4183–2.1784)	1
Unknown	62	53	9		
TMB					
<10	241	202	39	Reference	
≥10	60	38	22	2.9855 (1.5109–5.8432)	0.001
MSI					
MSS	265	221	44	Reference	
MSI‐H	30	15	15	4.985 (2.1047–11.8488)	1.00 E‐04
Unknown	6	4	2		

### 3.2 Genetic profiling of CRC

**FIGURE 1:**
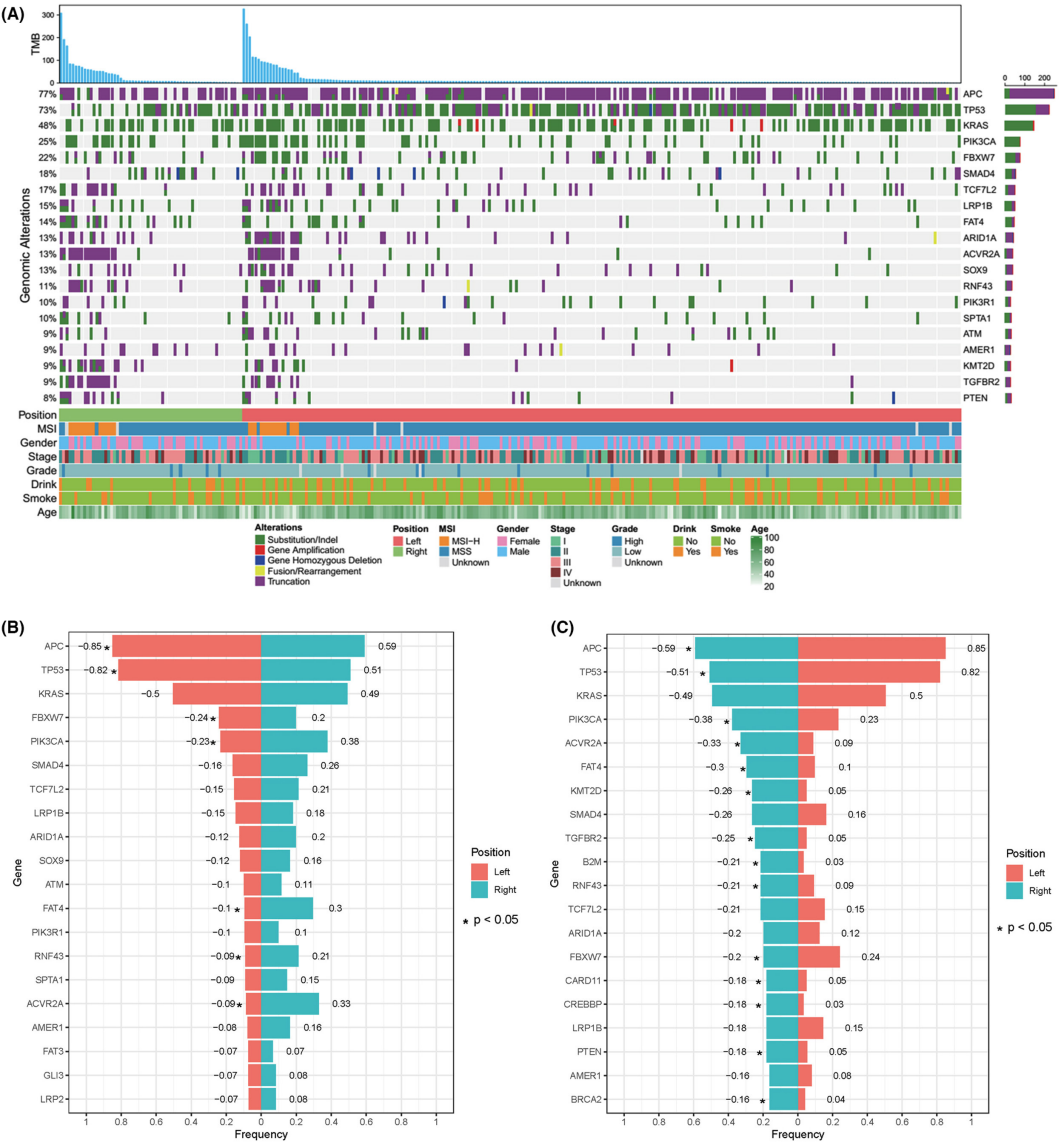
(A) The genomic variations of 301 CRCs. The panel shows the matrix of mutations colored by mutation type. The first row provides TMB values. Each column denotes an individual tumor and each row represents a gene. The right panel provides the gene name of the mutations and the left panel provides the proportion of mutations. Green: Substitution/Indel; Red: Gene amplification; Blue: Gene homozygous deletion; Yellow: Fusion/Rearrangement; Purple: Truncation. (B‐C) Genes with a significant difference in mutation frequency between left‐ and right‐sided CRC.

**FIGURE 2:**
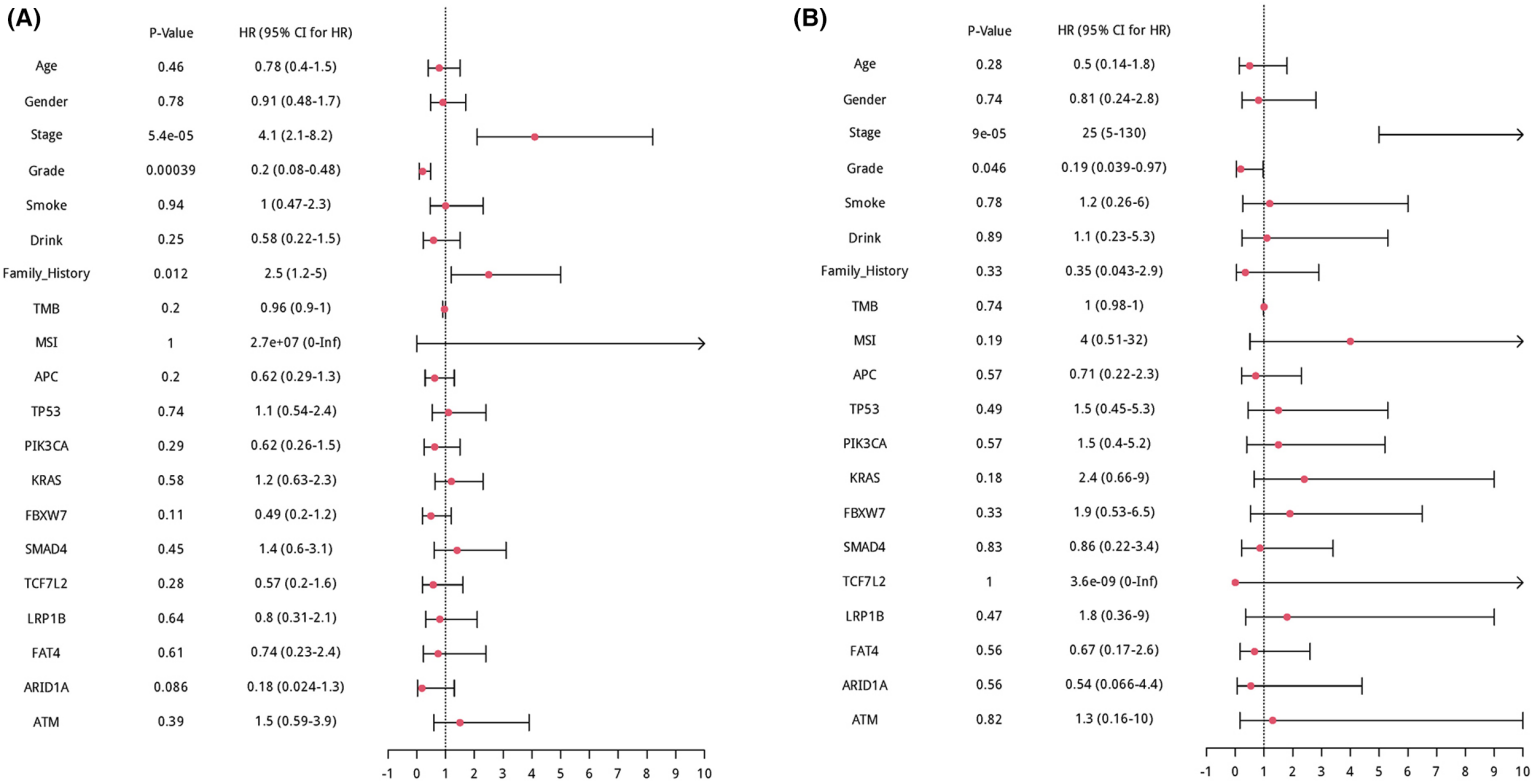
The multivariate Cox regression of CRC cohort. (A) Multivariate Cox regression of the left‐sided CRC. (B) Multivariate Cox regression of the right‐sided CRC.

Tumor samples from the 301 CRC patients were sequenced using NGS technology. Genetic profiling is provided in Figure 1A. A total of 2881 variations from 466 genes, including 1801 (62.51%) substitutions/indels, 252 (8.75%) gene amplifications, 779 (27.04%) truncations, 30 (1.04%) fusions/rearrangements, and 19 (0.66%) gene homozygous deletions were detected in the 301 CRC patients. The landscape of genetic alterations was mapped. The most common gene alterations for the 301 CRC patients were *APC* (77%), *TP53* (73%), *KRAS* (48%), *PIK3CA* (25%), *FBXW7* (22%), *SMAD4* (18%), *TCF7L2* (17%), *LRP1B* (15%), *FAT4* (14%), *ARID1A* (13%), *ACVR2A* (13%), *SOX9* (13%), *RNF43* (11%), *PIK3R1* (10%), and *SPTA1* (10%) (Figure 1A). The results of comutation analysis have shown in Figure S2. The most common gene alterations for the 80 right‐sided CRC patients and the 121 left‐sided CRC patients were also mapped (Tables S2 and S3 and Figure 1B,C, respectively). The *APC*, *TP53*, and *KRAS* genes were highly mutated in both left‐ and right‐sided CRCs. By comparing the mutation frequency of highly mutated genes, we found that the mutation frequency of *APC* and *TP53* in left‐sided CRC was significantly higher than that in right‐sided CRC, while the mutation frequency of *PIK3CA, ACVR2A, FAT4*, and *RNF43* in right‐sided CRC was significantly higher than that in left‐sided CRC. The multivariate Cox regression of the left‐sided and right‐sided CRC cohort was performed, respectively. In the right‐sided and left‐sided CRC cohort, stage, grade, age, gender, smoking history, drinking history, TMB, MSI status, and top high‐frequented mutated genes were included. The multivariate Cox regression showed that stage, grade, and family history were statistically significant independent prognostic factors in the left‐sided CRC (Figure 2A), and stage and grade were statically significant in the right‐sided CRC (Figure 2B).

### 3.3 DDR mutation landscape in MSS CRC patients

**FIGURE 3:**
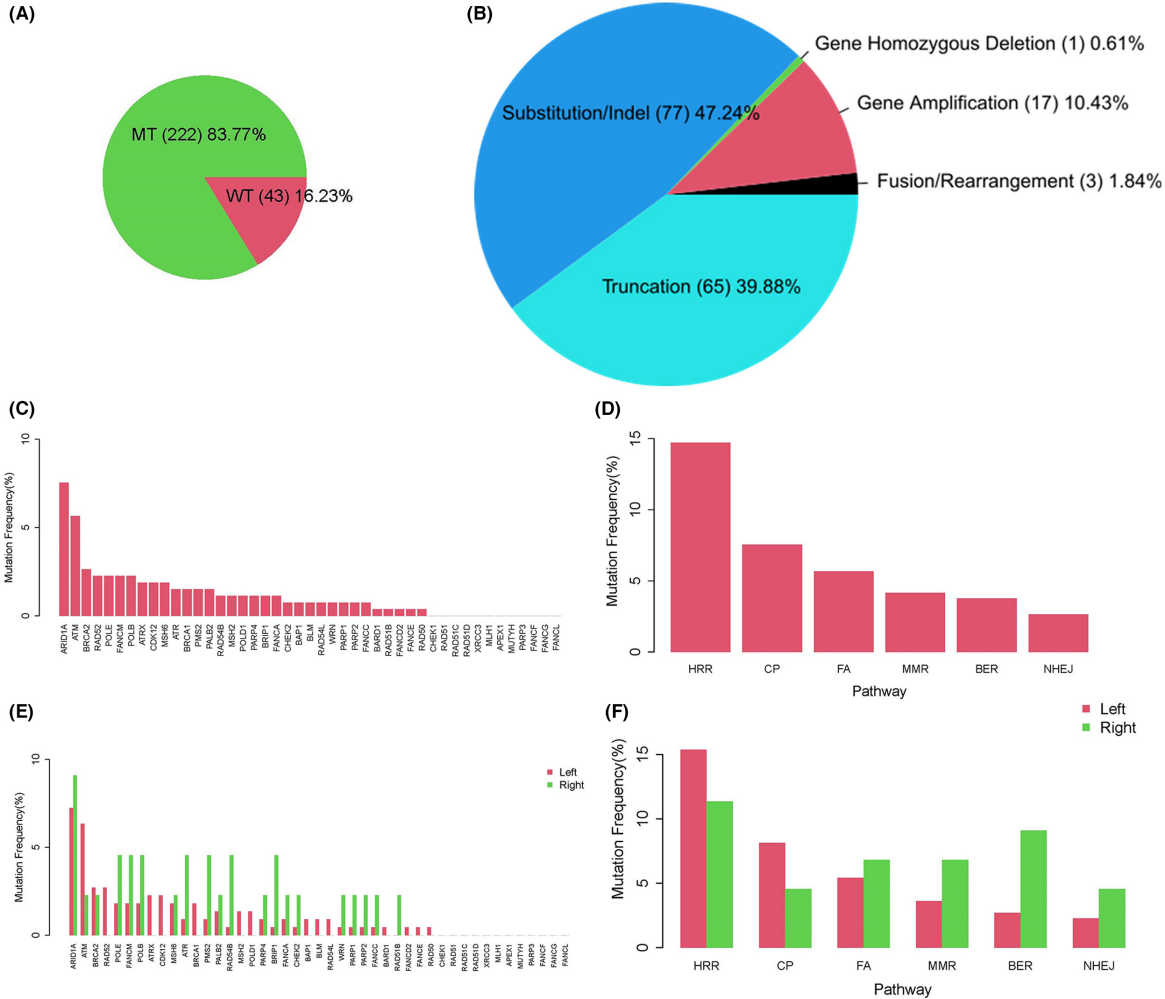
DDR mutations of MSS CRCs. (A) The mutation rate of DDR genes in MSS CRCs. (B) Gene mutation type and proportion. (C) DDR‐mutated genes and the mutation frequency of each DDR gene. (D) The mutations of DDR pathways and the mutation frequency of each DDR pathway. (E) A comparison of the mutational frequency of DDR genes between left‐ and right‐sided CRC. (F) A comparison of the mutational frequency of DDR pathways between left‐ and right‐sided CRC.

MSI‐H is significantly positively correlated with TMB‐H and is a known prognostic biomarker and immunotherapy biomarker in CRC. In our cohort, 30 CRC patients with MSI‐H all carried DDR mutations. Therefore, we excluded MSI‐H cases and analyzed the DDR mutation landscape in MSS CRC patients. Detailed characteristics of MSS CRC with DDR mutations are provided in Table 2. A total of 163 DDR gene somatic mutations, including 77 (47.24%) substitutions/indels, 65 (39.88%) truncations, 17 (10.43%) gene amplifications, 3 (1.84%) fusions/rearrangements, and 1 (0.61%) gene homozygous deletion were detected in 83.77% (222/265) of CRC patients (Figure 3A,B). Frequencies for every DDR gene mutation are summarized in Figure 2C. The most frequently mutated DDR genes were *ARID1A* (7.5%, 20/245), *ATM* (5.7%, 15/265), *BRCA2* (2.6%, 7/265), *PRD52* (2.3%, 6/259), *POLE* (2.3, 6/259), *FANCM* (2.3%, 6/259), and *POLB* (2.3%, 6/259) (Figure 3C). Frequencies of mutations in HRR, CP, FA, MMR, BER, NHEJ, and pathways were 14.72% (39/265), 7.5% (20/265), 5.7% (15/265), 4.1% (11/265), 3.8% (10/265), and 2.6% (7/265), respectively (Figure 3D). The frequency of mutated DDR genes and pathways was additionally compared between left‐ and right‐sided CRCs. As shown in Figure 3E,F, no significant difference was observed in DDR genes and pathways.

**TABLE 2:** Detailed characteristics of MSS CRC with DDR mutations.

	Total	DDR_MT	DDR_WT	OR (95% CI)	*p* value
Age					
≤55	97	81	16	Reference	
>55	168	141	27	0.9695 (0.4712–2.0486)	1
Gender					
Female	107	85	22	Reference	
Male	158	137	21	0.5935 (0.2909–1.2067)	0.1283
Stage					
I	29	22	7	Reference	
II	82	67	15	0.706 (0.2325–2.3209)	0.5888
III	107	90	17	0.5962 (0.2025–1.9156)	0.4091
IV	45	41	4	0.3119 (0.0601–1.3848)	0.0974
Unknown	2	2	0		
Grade					
Low	245	203	42	Reference	
High	16	15	1	0.3232 (0.0075–2.2144)	0.4836
Unknown	4	4	0		
Smoke					
No	202	164	38	Reference	
Yes	63	58	5	0.3732 (0.1094–1.0144)	0.0494
Drink					
No	208	174	34	Reference	
Yes	57	48	9	0.9597 (0.3781–2.2246)	1
Family history					
No	211	179	32	Reference	
Yes	49	39	10	1.4322 (0.5784–3.3042)	0.3905
Unknown	5	4	1		
Progress					
No	112	94	18	Reference	
Recurrent	4	3	1	1.731 (0.0314–22.9851)	0.5161
Metastatic	143	122	21	0.8993 (0.4288–1.9018)	0.8611
Unknown	6	3	3		
Survival					
Alive	161	133	28	Reference	
Dead	48	42	6	0.6797 (0.2153–1.8275)	0.5087
Unknown	56	47	9		
TMB					
<10	237	197	40	Reference	
≥10	28	25	3	0.592 (0.1092–2.0858)	0.5885

### 3.4 DDR mutation was not associated with clinical prognosis in MSS CRC

**FIGURE 4:**
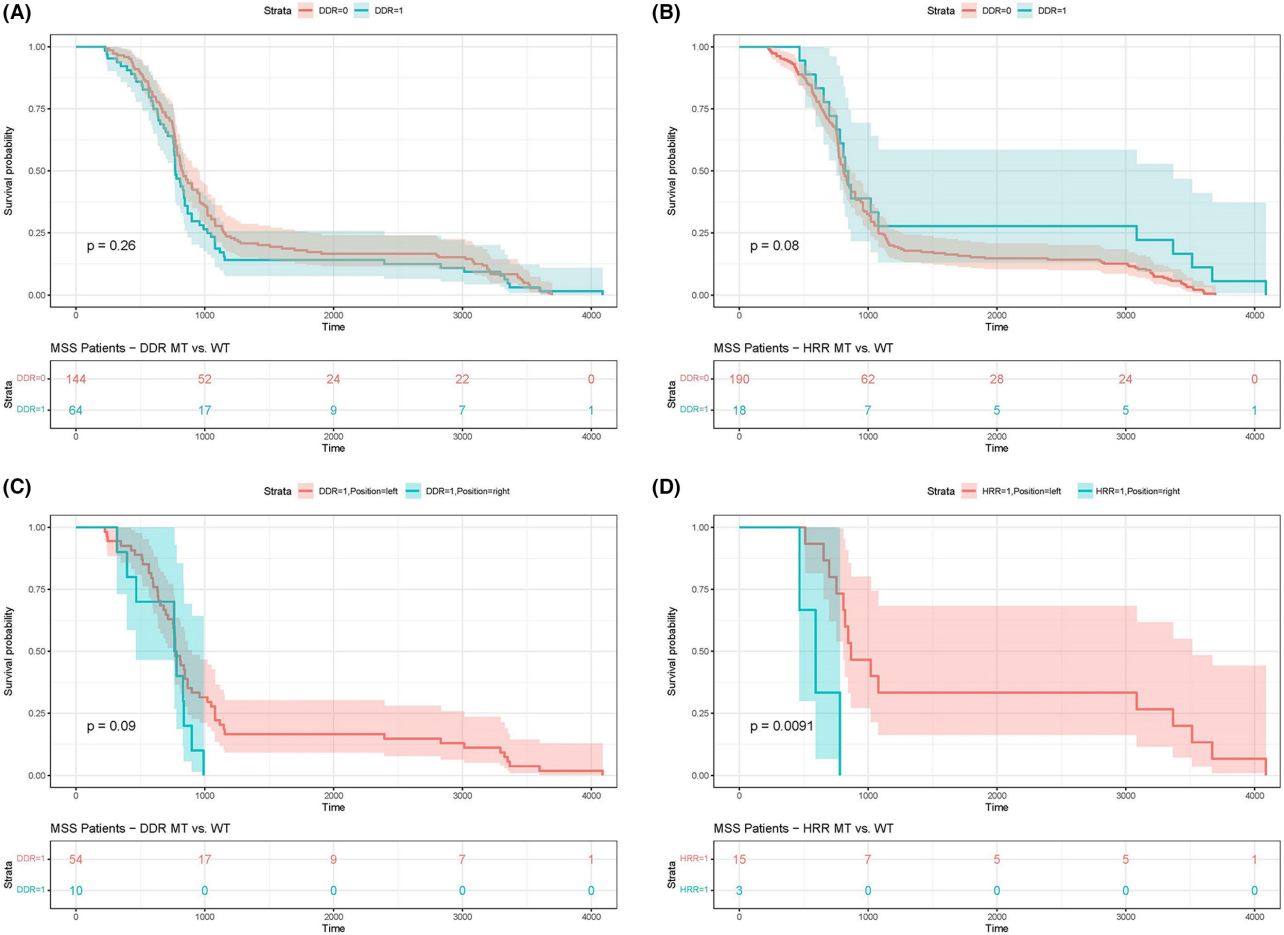
A correlation analysis of DDR mutations and prognosis. (A) DDR mutations in MSS CRC were not significantly related to OS. (B) HRR mutations in MSS CRC were not significantly related to OS. (C) No significant difference in OS among patients with DDR mutations in left‐ and right‐sided MSS CRC was determined. (D) Left‐sided MSS CRC patients with HRR mutations had a better OS compared with right‐sided MSS CRC.

We investigated whether or not DDR somatic mutations were associated with improved survival in MSS CRC patients. The presence of DDR somatic mutations was not significantly associated with better OS (*p* = 0.26) for MSS patients in our cohort (Figure 4A). Specifically, MSS patients with mutations in the HRR pathway did not display better OS (*p* = 0.08) (Figure 4B). Further analysis regarding left‐ and right‐sided CRC revealed no significant difference (*p* = 0.09) in OS between left‐ and right‐sided CRCs with DDR mutations (Figure 4C), whereas left‐sided CRC patients with HRR pathway mutations that were relatively independent of the *KRAS* mutation (*p* = 0.211), had a significantly prolonged OS compared with right‐sided CRC (*p* = 0.0091) (Figure 4D).

## 4 DISCUSSION

CRC is a highly heterogeneous malignancy with diverse clinical features, therapeutic responses, and prognosis. Therefore, identifying clinical or molecular biomarkers with predictive and prognostic values is necessary. In this study, we investigated the mutation landscape of 301 Chinese CRC patients and compared mutation profiles between left‐ and right‐sided CRC.

The genomic landscape of CRC has been well studied, and, in general, the genomic landscape of CRC remains relatively stable, with the most frequently mutated genes being *APC*, *TP53*, *KRAS*, *PIK3CA*, and *SMAD4*.^20^, ^21^ Our study further confirmed that the most common gene alterations for CRC patients are *APC* (77%), *TP53* (73%), *KRAS* (48%), *PIK3CA* (25%), *FBXW7* (22%), and *SMAD4* (18%). We additionally compared genetic mutation profiles between right and left‐sided CRC; and observed a higher mutation frequency for *APC* and *TP53* and a lower mutation frequency for *PIK3CA, ACVR2A, FAT4*, and *RNF43* in left‐sided CRC as compared to right‐sided CRC. Our results are highly consistent with a recent study which indicated that the mutation frequencies of *TP53* and *APC* in left‐sided CRC are significantly higher than that in right‐sided CRC, whereas the mutation frequency of *PIK3CA* is lower than that in right‐sided CRC.^22^, ^23^ *APC* encodes a tumor suppressor protein that combines with β‐catenin within the cytoplasm in the form of protein complexes and negatively regulates the β‐catenin and Wnt signaling pathways, thus preventing excessive cell proliferation.^24^ Different *APC* mutations lead to different levels of WNT/b‐catenin signaling pathway activation and are associated with the characteristics of different tumor sites in CRC.^25^ *TP53* is one of the most common tumor suppressor genes, both in CRC and in other tumor types.^26^ In CRC, mutations in *TP53* are associated with inferior survival.^27^ *PIK3CA* is involved in the PI3K/Akt signaling pathway and is associated with high mutation rates in CRC^28^; its somatic activating mutation also plays an important role during tumorigenesis.^29^ Enriched mutations of *TP53* and *APC* in left‐sided CRC and enriched mutations of *PIK3CA* in right‐sided CRC indicate the heterogeneity of CRC tumorigenesis and development.

In recent years, studies have revealed germline and/or DDR defects in CRC, with a prevalence between 13.8% and 36%.^13^, ^14^, ^15^ In our study, we identified 100% DDR mutations in MSI CRC and 83.77% in MSS CRC. Due to our inclusion of a greater number of DDR genes (45 DDR genes) compared with previous studies, we detected a higher DDR mutation rate. We further investigated the mutation frequency of DDR genes in MSS CRC and determined that the mutation incidence of *ARID1A* and *ATM* are notably higher than for other genes, consistent with the finding of alterations in *ARID1A* in 8.3% of CRCs^30^ and *ATM* in 7% of CRCs^31^ from previous studies. The most frequent mutation type, *ARID1A*, was a truncating mutation,^30^ like a frameshift mutation, that leads to DNA damage repair defects in tumor cells.^32^ Preclinical studies have shown that ARID1A deficiency sensitizes CRC cells to PARP inhibitors (olaparib, rucaparib, veliparib, or BMN673) in vitro and in vivo.^33^

A Phase II clinical trial (NCT02576444, OLAPCO) is currently ongoing for olaparil combination therapy in cancer patients with *PTEN, PIK3CA, AKT*, or *ARID1A* mutations or other mutations that lead to dysregulation of the PI3K/AKT pathway. *ATM* defects increase genomic instability by impeding the DNA double‐strand breakage (DSB) repair process but also increase tumor cell dependence on other DNA repair mechanisms, especially PARP‐mediated DNA single‐strand breakage (SSB).^34^, ^35^ Using the synthetic lethality mechanism, kinases (such as PARP) that inhibit the SSB repair process of *ATM*‐deficient tumors have potential therapeutic prospects.^34^, ^35^ Clinical trials are ongoing for several PARP inhibitors in patients with *ATM*‐deficient solid tumors (NCT01972217, NCT02693535, NCT03375307, NCT03233204, NCT03565991, and NCT03207347).

Agents targeting *ATMs* have drawn increasing attention from pharmaceutical companies.^36^ Recent research has indicated that *ALT* neuroblastoma chemotherapy resistance occurs via *ATM* activation and is reversible with the *ATM* inhibitor AZD0156. Combining AZD0156 with temozolomide plus irinotecan warrants clinical testing for neuroblastoma.^37^ Another *ATM* inhibitor, AZD1390, was verified to cross the intact blood–brain barrier, supporting the treatment of AZD1390 for glioblastoma multiforme or other brain malignancies.^38^ Targeted therapy for other DDR mutations, including *BRCA*, *ATR*, *ERCC2*, etc., is also in progress.^39^, ^40^, ^41^, ^42^ Our results indicate that targeted therapy, especially for *PARB* and *ATM* inhibitors, has great potential for the treatment of CRC harboring DDR mutations.

In addition to the DDR mutation landscape, we also analyzed the relationship between DDR mutations and clinical prognosis in MSS CRC. Our results revealed that DDR pathway mutations, including HRR pathway mutations, were not significantly associated with better OS in MSS CRC patients. Accordingly, Sebastian et al.^43^ found that DDR pathway alterations were not associated with survival or progression‐free survival (PFS) in CRC patients receiving oxaliplatin‐containing chemotherapy. Song et al.^13^ indicated that the DDR mutation was strongly associated with MSI status and was associated with a favorable median OS in CRC patients treated with ICI. However, in the Song et al.^13^ study, no significant difference was identified in the prognosis of patients with DDR mutations with conventional treatment, indicating that DDR mutations may be a specific biomarker for predicting the efficacy of ICI immunotherapy in CRCs. Therefore, for MSS CRC, it is reasonable that DDR mutations are not significantly associated with a better prognosis.

In our study, we observed that HRR pathway mutations were significantly associated with better OS in left‐sided MSS CRC patients compared with right‐sided MSS CRC patients. However, in our cohort, the number of left‐sided CRC patients with HRR mutations was much higher than that of right‐sided CRC patients (*n* = 15 vs. *n* = 3, respectively). As such, our data can only be used as a clinical reference. A larger sample size is needed for further validation.

In conclusion, we identified the most frequently mutated DDR genes: *ARID1A*, *ATM*, and *BRCA2* in CRC. Although DDR mutations do not significantly differ between left‐ and right‐sided CRC, and although no significant correlation exists between DDR mutations and prognosis in MSS CRC, we believe that DDR mutations remain a potential cancer therapeutic target for CRC treatment. MSS CRC still represents an unmet medical need. Going forward, how we can utilize DDR gene defects to expand treatment options and improve prognosis is an issue worth exploring.

## AUTHOR CONTRIBUTIONS

**Wei Huang:** Conceptualization (equal); project administration (equal); writing – original draft (equal). **Wenliang Li:** Conceptualization (equal); project administration (equal); writing – original draft (equal). **Ning Xu:** Methodology (equal); resources (equal); software (equal). **Hui Li:** Methodology (equal); resources (equal); validation (equal). **Zihan Zhang:** Formal analysis (equal); software (equal); visualization (equal). **Xiaolong Zhang:** Formal analysis (equal); methodology (equal); resources (equal). **Tingting He:** Data curation (equal); visualization (equal). **Jicheng Yao:** Formal analysis (equal); methodology (equal); software (equal); visualization (equal). **Mian Xu:** Writing – original draft (equal). **Qingqing He:** Formal analysis (equal). **Lijie Guo:** Formal analysis (equal); writing – review and editing (equal).

## FUNDING INFORMATION

This study was supported by the Yunnan Fundamental Research Projects (grant no. 2019FA039), the National Natural Science Foundation of China (grant no. 31660312), and leading medical talents in Yunnan (grant no. L‐2017001).

## Supporting information


Figure S1.

Figure S2.

Table S1–S3.

## Data Availability

The data that support the findings of this study are available upon request from the corresponding author. The data are not publicly available due to privacy or ethical restrictions.
