# Supplementary figures and images for: Differences in DNA damage repair gene mutations between left‐ and right‐sided colorectal cancer
Source: Cancer Med. 2023 Apr 25;12(9):10187–98. doi: 10.1002/cam4.5716 (PMC10225210; doi:10.1002/cam4.5716)

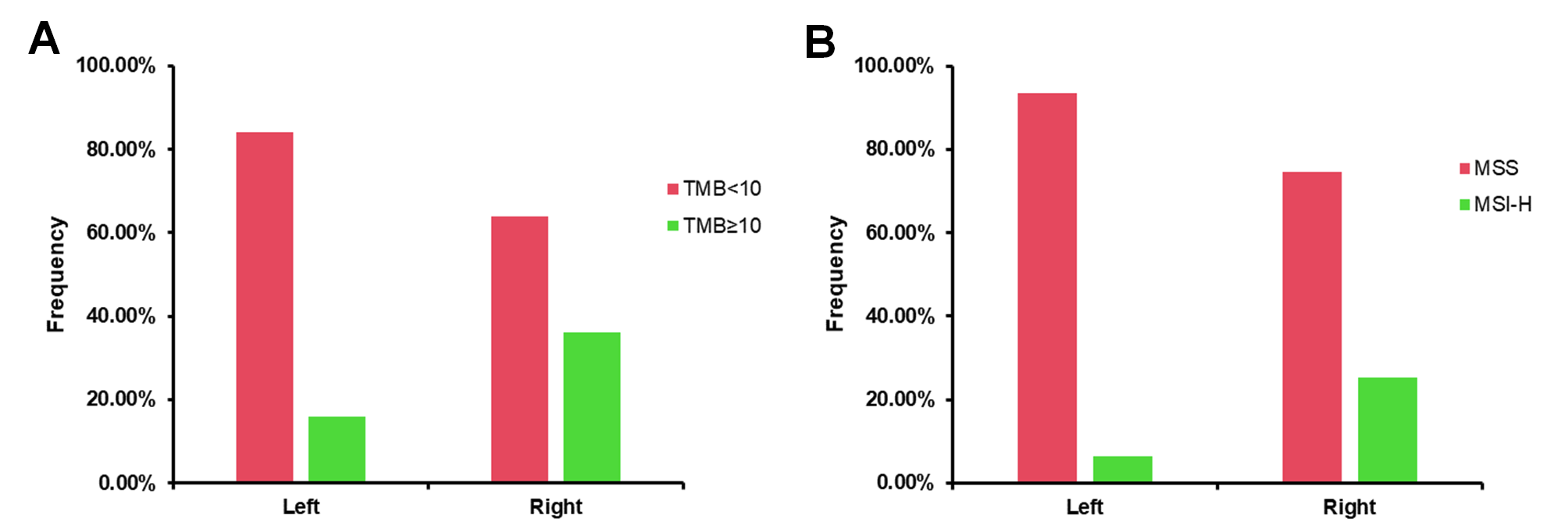

Supplement: Supplementary file 1 — Figure S1. [file CAM4-12-10187-s002.tiff]

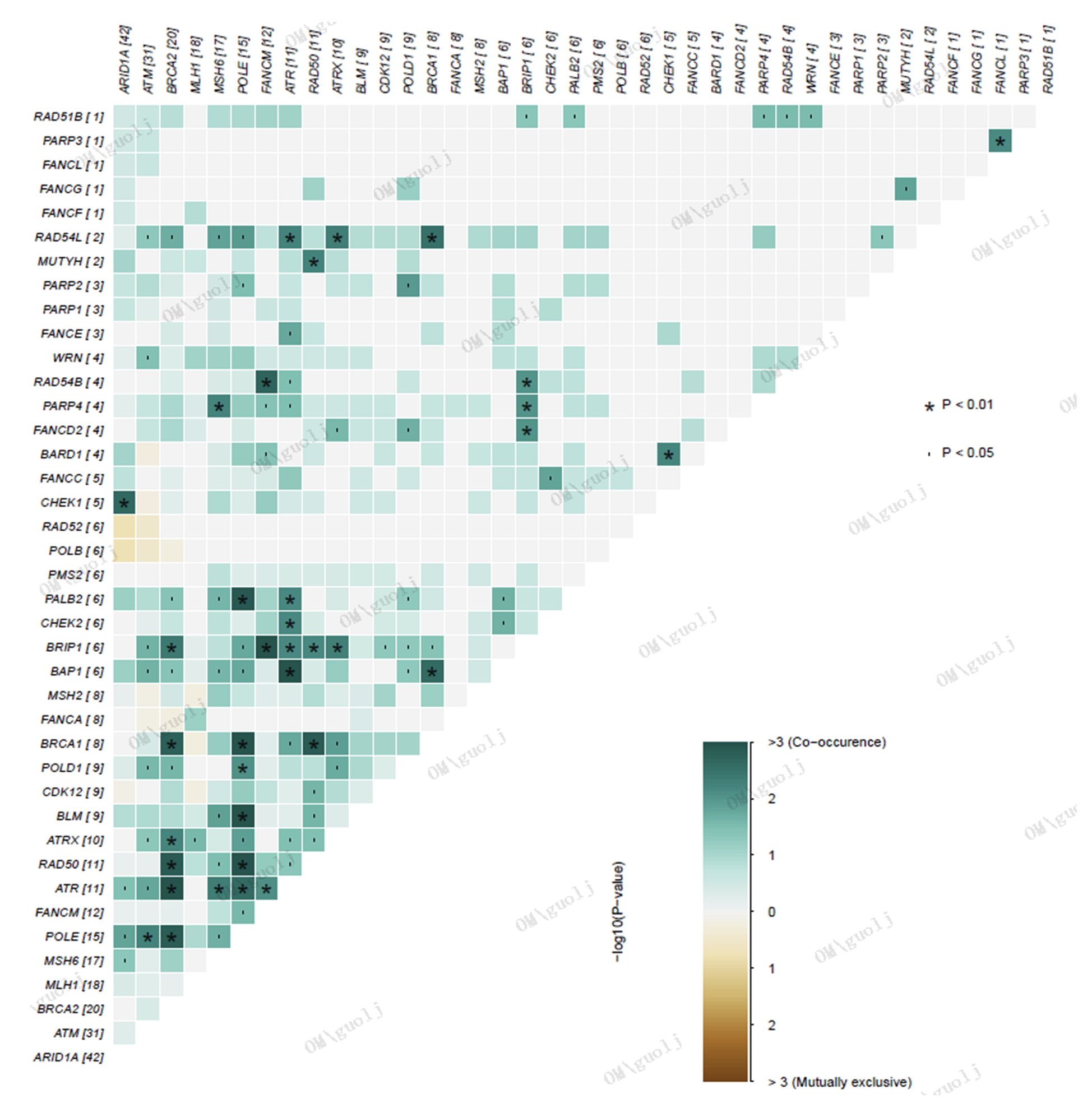

Supplement: Supplementary file 2 — Figure S2. [file CAM4-12-10187-s001.tiff]
