# Supplementary material for: Differences in DNA damage repair gene mutations between left‐ and right‐sided colorectal cancer
Source: Cancer Med. 2023 Apr 25;12(9):10187–98. doi: 10.1002/cam4.5716 (PMC10225210; doi:10.1002/cam4.5716)
Supplement: Supplementary file 3 — Table S1–S3. [file CAM4-12-10187-s003.docx]

Table S1. Forty-five DDR genes retrieved in this study

| **CPF 5** | **HRR 16** | **MMR 5** | **BER 7** | **FA 10** | **NHEJ 2** |
| --- | --- | --- | --- | --- | --- |
| ATM | ARID1A | MLH1 | APEX1 | BRIP1 | RAD50 |
| ATR | BAP1 | MSH2 | MUTYH | FANCA | POLB |
| ATRX | BARD1 | MSH6 | PARP1 | FANCC |  |
| CHEK1 | BLM | PMS2 | PARP2 | FANCD2 |  |
| CHEK2 | BRCA1 | POLD1 | PARP3 | FANCE |  |
|  | BRCA2 |  | PARP4 | FANCF |  |
|  | RAD51 |  | POLE | FANCG |  |
|  | RAD51B |  |  | FANCL |  |
|  | RAD51C |  |  | FANCM |  |
|  | RAD51D |  |  | PALB2 |  |
|  | RAD54B |  |  |  |  |
|  | RAD54L |  |  |  |  |
|  | WRN |  |  |  |  |
|  | CDK12 |  |  |  |  |
|  | XRCC3 |  |  |  |  |
|  | RAD52 |  |  |  |  |

Table S2 The top20 gene mutations in the left sided CRC, and the mutation frequency of these genes on the right sided CRC.

| Gene | Frequency-Left | Frequency-Right |
| --- | --- | --- |
| APC | 85% | 59% |
| TP53 | 82% | 51% |
| KRAS | 50% | 49% |
| FBXW7 | 24% | 20% |
| PIK3CA | 23% | 38% |
| SMAD4 | 16% | 26% |
| TCF7L2 | 15% | 21% |
| LRP1B | 15% | 18% |
| ARID1A | 12% | 20% |
| SOX9 | 12% | 16% |
| ATM | 10% | 11% |
| FAT4 | 10% | 30% |
| PIK3R1 | 10% | 10% |
| RNF43 | 9% | 21% |
| SPTA1 | 9% | 15% |
| ACVR2A | 9% | 33% |
| AMER1 | 8% | 16% |
| FAT3 | 7% | 7% |
| GLI3 | 7% | 8% |
| LRP2 | 7% | 8% |

Table S3 The top20 gene mutations in the right sided CRC, and the mutation frequency of these genes on the left sided CRC.

| Gene | Frequency- Right | Frequency- Left |
| --- | --- | --- |
| APC | 59% | 85% |
| TP53 | 51% | 82% |
| KRAS | 49% | 50% |
| PIK3CA | 38% | 23% |
| ACVR2A | 33% | 9% |
| FAT4 | 30% | 10% |
| KMT2D | 26% | 5% |
| SMAD4 | 26% | 16% |
| TGFBR2 | 25% | 5% |
| B2M | 21% | 3% |
| RNF43 | 21% | 9% |
| TCF7L2 | 21% | 15% |
| ARID1A | 20% | 12% |
| FBXW7 | 20% | 24% |
| CARD11 | 18% | 5% |
| CREBBP | 18% | 3% |
| LRP1B | 18% | 15% |
| PTEN | 18% | 5% |
| AMER1 | 16% | 8% |
| 16 | 16% | 4% |
